# Successful Endoscopic Closure of Late Ileal Pouch Stump Perforation Using Conventional Through-the-Scope Clips After Ileal Pouch-Anal Anastomosis: A Case Report

**DOI:** 10.7759/cureus.112719

**Published:** 2026-07-15

**Authors:** Kazuhito Minami, Hiroko Yano, Ryosuke Kiyomori, Koji Ikegami, Koichi Kurahara

**Affiliations:** 1 Surgery, Matsuyama Red Cross Hospital, Matsuyama, JPN; 2 Surgery, Matsue Red Cross Hospital, Matsuyama, JPN; 3 Gastroenterology, Hamanomachi Hospital, Fukuoka, JPN; 4 Gastroenterology, Matsue Red Cross Hospital, Matsuyama, JPN

**Keywords:** anastomotic stricture, familial adenomatous polyposis, ileal pouch-anal anastomosis, ileal pouch stump perforation, through-the-scope clips

## Abstract

Ileal pouch stump perforation is an extremely rare late complication after ileal pouch-anal anastomosis (IPAA), and most reported cases have required operative management. Endoscopic closure with conventional through-the-scope (TTS) clips has rarely been described in this setting, particularly when advanced closure devices are limited by anatomy. A 50-year-old man with familial adenomatous polyposis (FAP) presented with lower abdominal pain seven years after total proctocolectomy with handsewn IPAA. Computed tomography (CT) showed a localized pelvic abscess and small foci of free air adjacent to the ileal pouch stump, consistent with localized perforation. Because he was hemodynamically stable and had no generalized peritonitis, initial conservative treatment was performed. Fluoroscopy-assisted pouchoscopy identified a small stump defect with localized contrast leakage. Over-the-scope clip deployment was considered unsafe because of a severe pouch-anal anastomotic stricture; therefore, conventional TTS clips were applied in a zipper fashion. Recurrent leakage on planned second-look endoscopy was treated successfully with additional TTS clips. Complete closure was confirmed endoscopically and radiologically, and no recurrence was observed during 10 months of follow-up. This case suggests that, in carefully selected stable patients with localized ileal pouch stump perforation and a small identifiable defect, repeated conventional TTS clip closure after initial infection control may be a practical minimally invasive alternative to surgery when over-the-scope clip delivery is technically difficult.

## Introduction

Ileal pouch-anal anastomosis (IPAA) is a standard restorative procedure after total proctocolectomy for familial adenomatous polyposis (FAP) and ulcerative colitis [1,2]. Well-recognized late complications include pouchitis, anastomotic stricture, and functional disorders, whereas perforation of the ileal pouch stump is extremely rare, with fewer than 15 cases documented in the literature worldwide [3-13]. Reported cases have typically required operative management, such as stump repair or resection, intra-abdominal drainage, and/or diverting ileostomy [4-13]. However, evidence regarding optimal management strategies for spontaneous pouch stump perforation remains limited, and the literature provides little guidance for minimally invasive approaches.

We report a patient with FAP who developed late ileal pouch stump perforation seven years after IPAA and was successfully treated without surgery using conventional through-the-scope (TTS) clips following initial conservative management. This case highlights a step-up, minimally invasive strategy for selected stable patients with localized inflammation and a small identifiable defect. Such an approach may be particularly useful when the bulkier over-the-scope clip (OTSC) delivery system cannot pass through a narrow stricture, making the slimmer TTS clips a feasible alternative. This report underscores both the rarity of this complication and the potential role of endoscopic therapy in carefully chosen patients.

## Case presentation

A 50-year-old man had been diagnosed with FAP at 22 years of age. Seven years before presentation, he underwent laparoscopic total proctocolectomy with handsewn IPAA and diverting loop ileostomy, followed by ileostomy closure four months later. Surveillance endoscopy performed six years after IPAA revealed a tight pouch-anal anastomotic stricture through which only a thin endoscope could pass with difficulty. Because he had no obstructive symptoms and no preceding risk factors such as pouchitis, trauma, medication changes, or recent endoscopic intervention, he was followed conservatively without dilation.

Seven years after IPAA, he presented to our emergency department as a walk-in with several days of persistent lower abdominal pain. His vital signs were stable, and he was afebrile. Abdominal examination showed localized lower abdominal tenderness without rebound tenderness or guarding, indicating the absence of generalized peritonitis. Laboratory testing demonstrated leukocytosis, elevated C-reactive protein (CRP), and elevated aminotransferase levels. The elevated aminotransferases were considered unrelated to the perforation and were attributed to his habitual alcohol consumption (approximately 5 liters of beer per week) and possible increased intake immediately before symptom onset (Table 1).

**Table 1:** Laboratory findings upon admission Note: Elevated aminotransferases (aspartate aminotransferase/alanine aminotransferase) may have been related to habitual alcohol consumption and possible heavy intake before symptom onset.

Parameter	Value	Reference values
White blood cell	10,740/μL	3,300-8,600/μL
Hemoglobin	14.4 g/dL	13.7-16.8 g/dL
Platelet	29.9 × 10⁴/μL	15.8-34.8 × 10⁴/μL
Total protein	7.6 g/dL	6.7-8.3 g/dL
Total bilirubin	0.8 mg/dL	0.2-1.2 mg/dL
Albumin	4.0 g/dL	3.8-5.3 g/dL
Aspartate aminotransferase	173 U/L	13-30 U/L
Alanine aminotransferase	172 U/L	10-42 U/L
Lactate dehydrogenase	232 U/L	124-222 U/L
Blood urea nitrogen	11.4 mg/dL	8-20 mg/dL
Creatinine	0.97 mg/dL	0.65-1.07 mg/dL
Na	140 mmol/L	138-145 mmol/L
K	4.5 mmol/L	3.6-4.8 mmol/L
Cl	103 mmol/L	101-108 mmol/L
C-reactive protein	8.42 mg/dL	0-0.14 mg/dL
Fasting blood sugar	98 mg/dL	100-109 mg/dL

**Figure 1:**
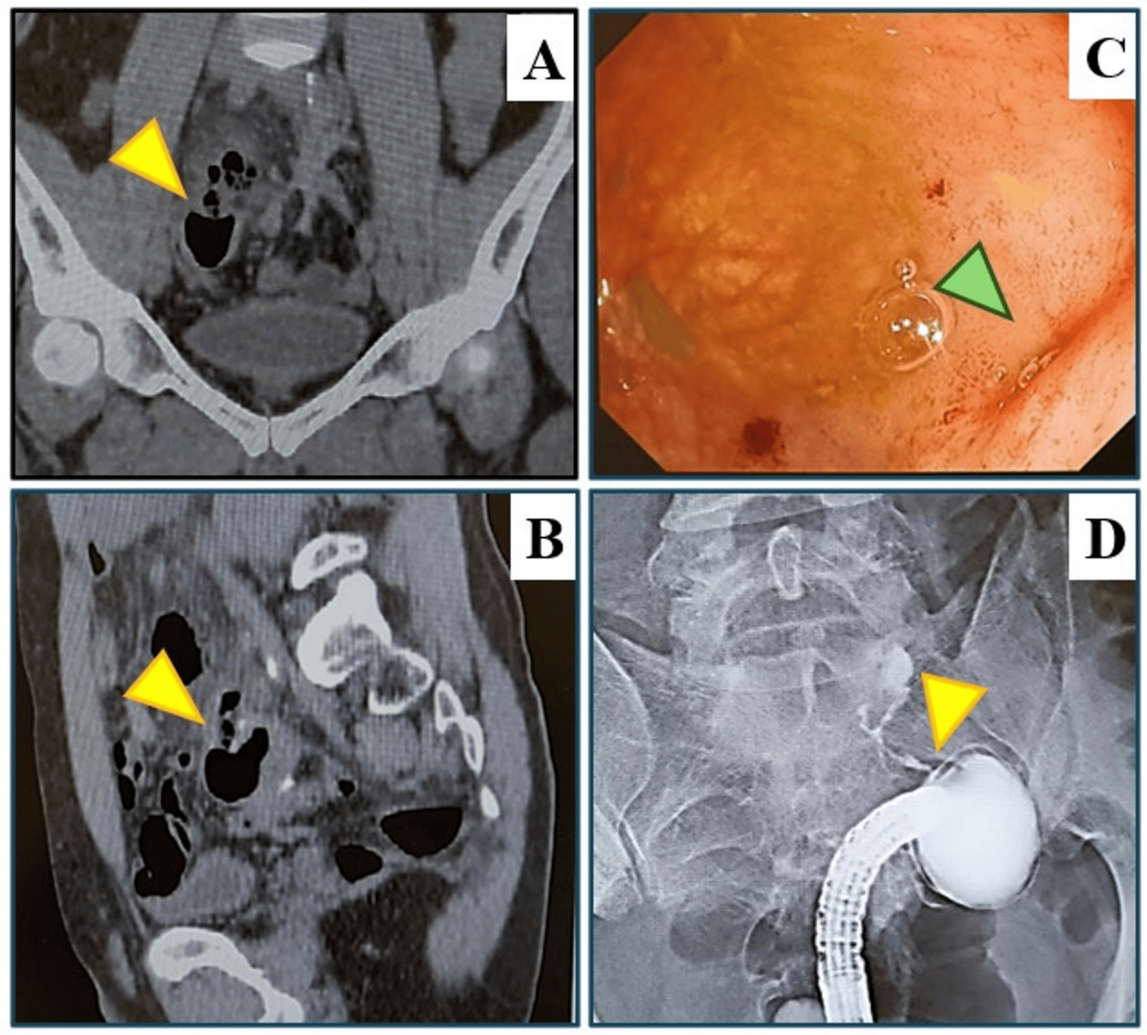
Initial imaging and fluoroscopy-assisted endoscopic findings Contrast-enhanced CT showed a localized pelvic abscess adjacent to the ileal pouch stump, or blind end, with small foci of free air, suggesting ileal pouch stump perforation and localized peritonitis (A (coronal view) and B (sagittal view)). Fluoroscopy-assisted pouchoscopy identified a small fistulous opening at the blind end with surrounding erythema (C). Contrast injection demonstrated localized extravasation into the abscess cavity (D). Yellow arrowheads indicate the suspected perforation site or contrast leakage, and the green arrowhead indicates the endoscopically identified fistulous opening. CT: computed tomography

Contrast-enhanced computed tomography (CT) revealed a localized pelvic abscess adjacent to the ileal pouch stump with small foci of free air, consistent with pouch stump perforation and localized peritonitis (Figure 1A, 1B).

Because there were no signs of sepsis or generalized peritonitis, non-operative management was initiated with bowel rest, nil per os, intravenous antibiotics, and close clinical monitoring. He received intravenous meropenem at 3 g/day, administered for four days initially, with subsequent re-administration for six days when inflammatory markers rose again. His abdominal pain and inflammatory markers gradually improved; by hospital day (HD) 5, his white blood cell (WBC) had decreased to 4,520/μL and CRP to 1.88 mg/dL, supporting the decision to proceed with fluoroscopy-assisted pouchoscopy, which identifies the perforation site and assesses the feasibility of endoscopic closure [14,15]. Endoscopy confirmed a tight pouch-anal anastomotic stricture. At the ileal pouch stump, a small mucosal defect with surrounding erythema was identified, and contrast injection demonstrated localized leakage into the abscess cavity (Figure 1C, 1D).

Closure using an OTSC system was initially considered. However, the therapeutic endoscopist judged that safe delivery of the bulkier OTSC system through the anal canal and tight pouch-anal anastomotic stricture would be technically difficult, even with prior bougie dilation. In contrast, conventional TTS clips, delivered through the working channel of the endoscope, could be advanced beyond the stricture, making them a feasible alternative [14,15].

**Figure 2:**
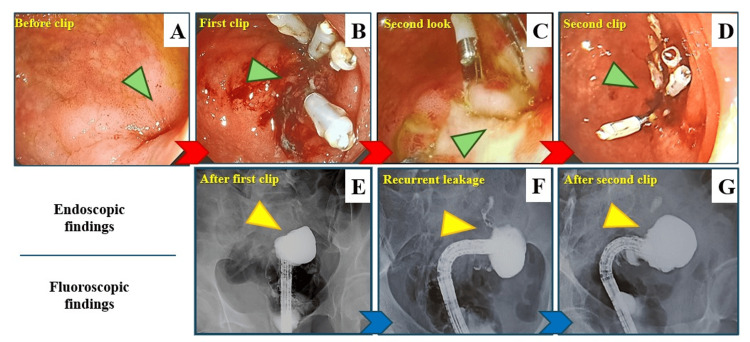
Endoscopic closure Before clipping, endoscopy showed a small fistulous opening at the ileal pouch stump (A). On HD 8, multiple conventional TTS clips were applied in a zipper fashion to approximate the defect margins (B), and immediate fluoroscopic contrast injection confirmed disappearance of leakage (E). On HD 11, second-look endoscopy showed reopening of the defect (C), and fluoroscopy demonstrated recurrent contrast leakage (F). Additional clips were applied in a zipper fashion (D), after which fluoroscopy confirmed disappearance of leakage (G). The green arrowhead marks the fistula identified on endoscopy during both its open and subsequently closed states. The yellow arrowhead indicates the fistulous site visualized on fluoroscopy in both its open and later closed phases. HD: hospital day, TTS: through-the-scope

On HD 8, the first endoscopic closure was attempted. A small fistulous opening at the ileal pouch stump was identified, and four standard Olympus TTS clips were sequentially applied in a zipper fashion to approximate the defect margins. Immediate fluoroscopic contrast injection confirmed disappearance of leakage (Figure 2A, 2B, 2E). On HD 11, planned second-look endoscopy with fluoroscopy demonstrated reopening of the defect and recurrent contrast leakage from the same site. Two clips were found to have detached, and four additional Olympus TTS clips were applied in a zipper fashion, again resulting in disappearance of leakage on fluoroscopic examination (Figure 2C, 2F, 2D, 2G).

**Figure 3:**
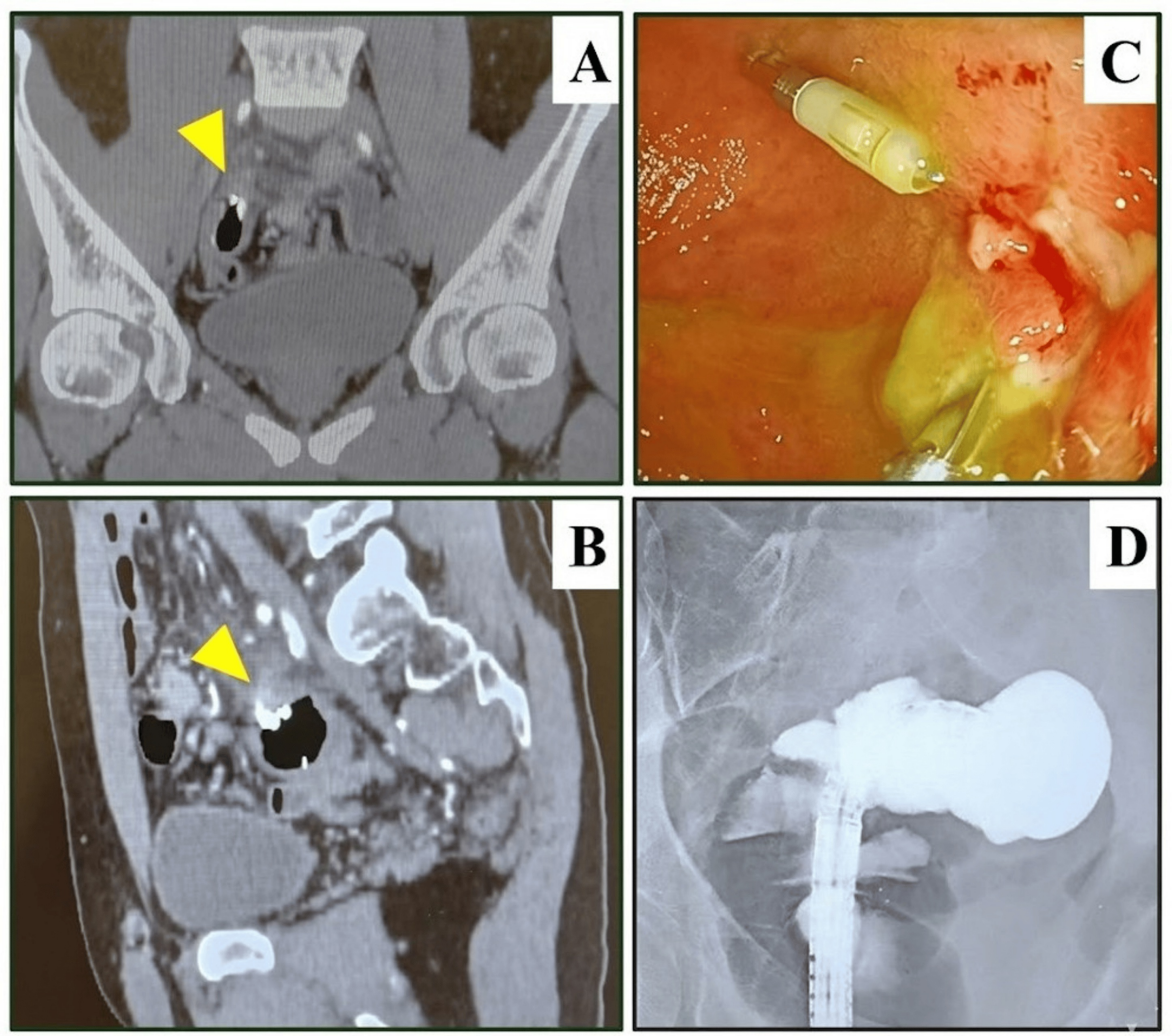
Confirmation of healing On HD 17, follow-up CT showed resolution of the pelvic abscess and deployed clips at the perforation site (A (coronal view) and B (sagittal view)). Endoscopy showed the clipped defect without an open fistula (C), and fluoroscopic contrast examination confirmed complete closure without residual leakage (D). Yellow arrowheads indicate the deployed endoscopic clips at the perforation site in A and B. HD: hospital day, CT: computed tomography

On HD 17, follow-up CT showed resolution of the pelvic abscess, endoscopy showed no open fistula, and fluoroscopic contrast examination confirmed complete closure without residual leakage (Figure 3A-3D).

**Figure 4:**
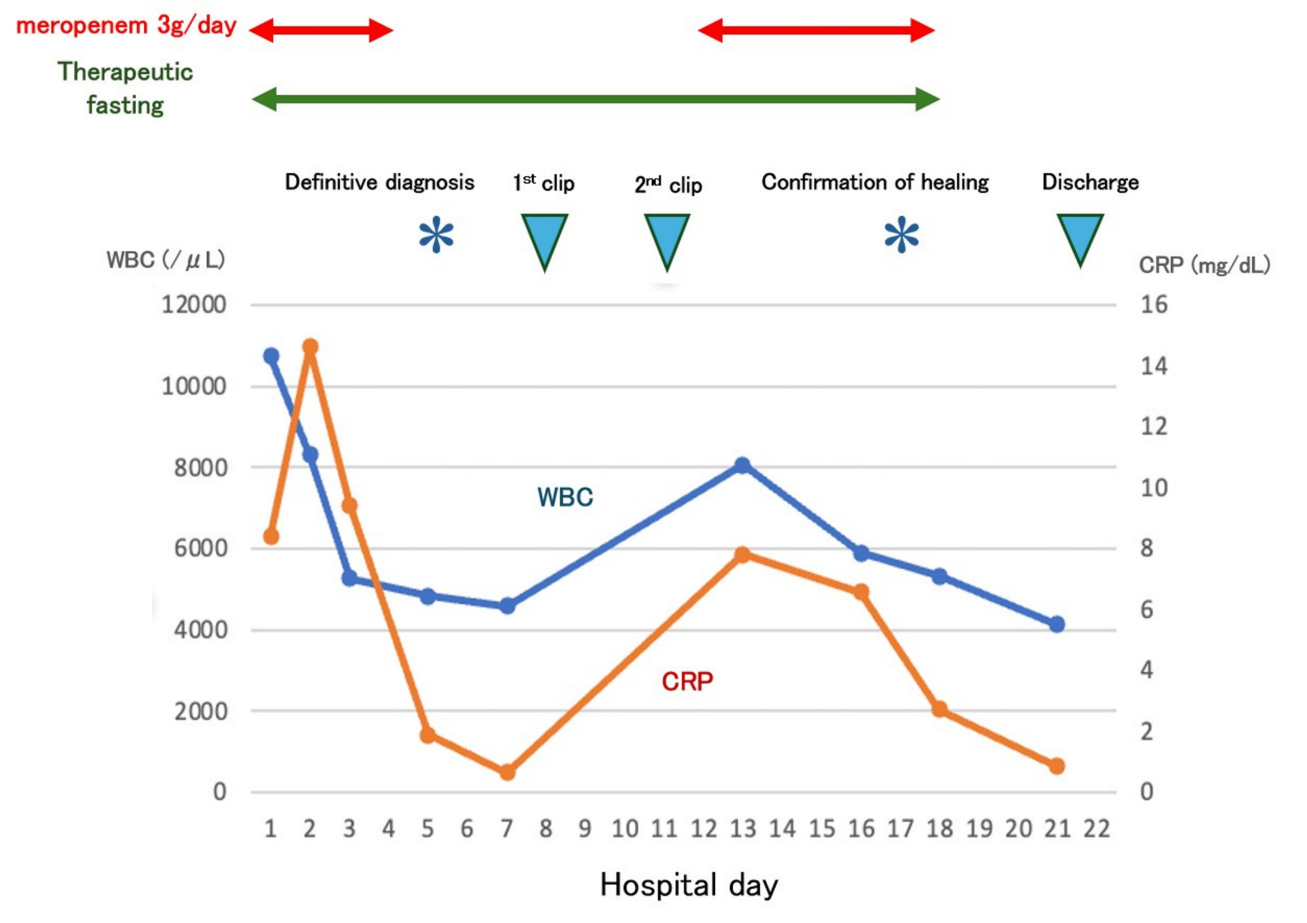
Clinical course Serial changes in inflammatory markers during hospitalization. The blue line represents the WBC count, and the orange line represents the CRP level. The left and right Y-axes indicate the respective scales for WBC and CRP. The X-axis shows HD. WBC and CRP gradually decreased after initiation of conservative management with meropenem and therapeutic fasting. The first endoscopic closure using TTS clips was performed on HD 8, followed by a second closure on HD 11 after reopening of the defect. Radiologic and endoscopic confirmation of complete healing was achieved on HD 17, after which oral intake was resumed. The patient was discharged on HD 22 with sustained improvement in inflammatory markers. WBC: white blood cell, CRP: C-reactive protein, HD: hospital day

Oral intake was resumed after closure was confirmed. During hospitalization, gentle dilation of the pouch-anal anastomotic stricture　(≈10 mm in diameter) was performed using a single finger (≈15 mm in diameter) as a finger bougie. This resulted in improvement of the luminal narrowing. He was discharged on HD 22 without complications. Figure 4 summarizes the overall clinical course and endoscopic management during hospitalization.

**Figure 5:**
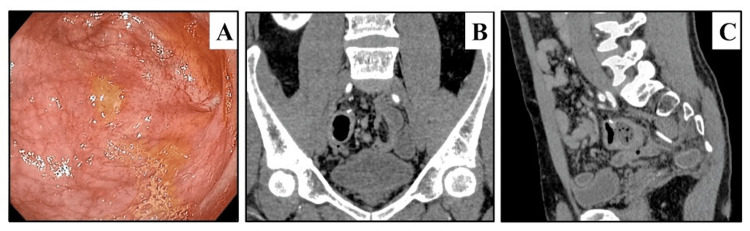
Ten-month follow-up after endoscopic closure Endoscopy showed complete mucosal healing at the ileal pouch stump without recurrent fistula or ulceration (A). Contrast-enhanced CT showed no residual pelvic abscess, free air, or inflammatory changes around the pouch stump (B (coronal view) and C (sagittal view)), confirming durable resolution. CT: computed tomography

At 10-month follow-up, he remained asymptomatic; endoscopy showed complete healing of the pouch stump defect, and CT demonstrated resolution of the pelvic abscess without recurrence (Figure 5A-5C).

## Discussion

Ileal pouch stump perforation after IPAA is an extremely rare late complication. Previously reported cases have typically presented years after IPAA and have generally required surgery, including repair or resection of the perforated stump, intra-abdominal drainage, and/or diverting ileostomy [4-13]. Several precipitating factors have been proposed, including pouch distension, pouch outlet obstruction, anastomotic stricture, volvulus or torsion of the pouch tip, infectious enteritis, trauma, pregnancy, and acute increases in intraluminal pouch pressure (Table 2).

**Table 2:** Reported cases of ileal pouch stump perforation after IPAA/RPC UC: ulcerative colitis, FAP: familial adenomatous polyposis, IPAA: ileal pouch-anal anastomosis, RPC: restorative proctocolectomy, NR: not reported, y: years, m: months, TTS: through-the-scope, F: female, M: male

Author (year)	Sex	Indication	Suspected cause/trigger	Interval from IPAA	Site	Treatment
Lontoft (1986) [6]	F	UC	Salmonella typhimurium infection	2 y 6 m	NR	NR
Pezim et al. (1987) [7]	M	UC	Volvulus/torsion of terminal ileal appendage after J-pouch construction	3 y 5 m	Terminal ileal appendage/reservoir-related	NR
Hsu (1989) [8]	F	UC	Blunt abdominal trauma	NR	Afferent loop of pouch	Laparotomy; debridement and primary closure; temporary pouch decompression
Shapiro et al. (2000) [9]	F	UC	Rapid intake of high-fiber/high-calorie meal (hypothesized acute pressure elevation/obstruction)	2 y	Distal transection site	NR
Aouthmany and Horattas (2004) [10]	F	UC	Pregnancy; adhesions between pouch and gravid uterus	10 y	NR	Emergency laparotomy and cesarean section (repair details NR)
Takahashi et al. (2009) [4]	F	UC	Enlarged blind end and pouchitis	8 y	Blind end	Laparotomy; resection of blind end
Dogan et al. (2015) [11]	M	FAP	Pouchitis/pouch dilation	5 y	Blind end	Laparotomy; primary repair and protective ileostomy
Panwar et al. (2016) [12]	F	UC	Idiopathic spontaneous perforation	1 y	NR	Emergency laparotomy; primary closure, lavage, and diverting loop ileostomy
Drober et al. (2021) [13]	M	UC	Blunt abdominal trauma	20 y	Blind end	Exploratory laparotomy; primary closure and diverting loop ileostomy
Shibata et al. (2022) [5]	M	FAP	High intraluminal pressure related to anastomotic stricture (first event)	6 y	Blind end	Blind-end resection, abdominal drainage, and anastomotic dilation
Shibata et al. (2022) [5]	M	FAP	High intraluminal pressure related to anastomotic stricture (recurrent event)	18 y	Blind end	Laparotomy; repair and temporary loop ileostomy
Present case	M	FAP	Anastomotic stricture with elevated pouch pressure	7 y	Blind end stump	Conservative therapy followed by endoscopic closure with conventional TTS clips (no surgery)

In the present case, the longstanding pouch-anal anastomotic stricture was considered the main local factor contributing to perforation at the structurally vulnerable blind end. Although mild localized erythema was observed around the defect, the patient had no clinical features suggestive of pouchitis, and occult pouchitis was therefore considered unlikely to be the primary cause.

From a mechanistic standpoint, several factors may contribute to delayed pouch stump perforation. Chronic mechanical stress from persistent outlet obstruction, intermittent episodes of elevated intraluminal pressure, localized ischemia at the blind end, and structural weakness of the pouch wall may all play roles. Although the present case lacked overt inflammatory disease, chronic low-grade inflammation cannot be entirely excluded. These mechanisms should be considered when evaluating patients with late-onset pouch-related complications.

The key management decision in this case was to avoid immediate surgery and adopt a step-up strategy. This approach was appropriate because the patient was hemodynamically stable, had localized peritonitis without sepsis or generalized peritoneal signs, and demonstrated sufficient improvement with conservative treatment to allow clear endoscopic identification of the defect under fluoroscopic guidance. In this setting, endoscopic closure was considered a reasonable minimally invasive option prior to surgical intervention [14,15].

OTSC systems are useful for closing gastrointestinal perforations and leaks because they provide strong tissue approximation. However, their delivery can be limited by anatomical constraints, particularly in patients with severe strictures or difficult access routes [14,15]. In this patient, safe passage of the OTSC system through the anal canal and tight pouch-anal anastomotic stricture was judged to be technically difficult. Conventional TTS clips were therefore selected because they could be delivered through the endoscope beyond the stricture. Although TTS clips generally provide less closing force than OTSC systems, closure was achieved by sequentially approximating the defect margins in a zipper fashion under fluoroscopic guidance.

Planned reassessment was another essential component of this strategy. Immediate disappearance of contrast leakage after clipping did not guarantee durable closure, as recurrent leakage was detected on second-look endoscopy. Additional clipping resulted in complete closure, suggesting that repeated endoscopic and fluoroscopic evaluation may be useful when a conservative endoscopic approach is selected. Gentle dilation of the pouch-anal anastomotic stricture was also performed using a finger bougie technique, which contributed to improved pouch emptying and reduced pressure load.

Differential diagnosis for lower abdominal pain in IPAA patients includes pouchitis, cuffitis, pelvic abscess, anastomotic leak, volvulus of the pouch tip, and small bowel obstruction. In this case, the absence of systemic inflammation, lack of endoscopic features of pouchitis, and localized CT findings helped narrow the diagnosis to localized pouch stump perforation.

This case also underscores the importance of long-term surveillance after IPAA. Follow-up should include not only neoplastic surveillance but also assessment and timely management of anastomotic stricture, which may contribute to pressure-related late complications. Early identification and treatment of outlet obstruction may help prevent similar events.

This report has limitations. It describes a single patient, and the approach should not be generalized to patients with sepsis, generalized peritonitis, large defects, uncontrolled abscesses, or defects that cannot be clearly identified endoscopically. Nevertheless, the case demonstrates that conventional TTS clip closure can be a practical salvage option when OTSC deployment is technically difficult and the clinical situation permits a step-up strategy. The treatment strategy described here may serve as a useful minimally invasive option in carefully selected patients.

## Conclusions

Ileal pouch stump perforation is a rare late complication after IPAA and has traditionally required surgical management. In selected stable patients with localized peritonitis and a clearly identifiable small defect, conventional TTS clip closure after initial conservative treatment may serve as a feasible minimally invasive alternative to surgery, particularly when OTSC deployment is limited by severe anastomotic stricture. Careful follow-up and timely recognition and management of pouch-anal anastomotic stricture may help reduce intraluminal pressure and prevent pressure-related late complications after IPAA.
